# Examining the characteristics of patients with long-term impaired work ability in primary health care – a cross-sectional study

**DOI:** 10.1080/02813432.2026.2638516

**Published:** 2026-03-23

**Authors:** Märit Löfgren, Lena Nordeman, Nashmil Ariai, Cecilia Björkelund, Gun Rembeck, Irene Svenningsson, Karin Törnbom, Dominique Hange

**Affiliations:** a Primary Health Care/School of Public Health and Community Medicine, Institute of Medicine, The Sahlgrenska Academy, University of Gothenburg, Gothenburg, Sweden; b Region Västra Götaland, Research, Education, Development & Innovation Primary Health Care, Research, Education, Development & Innovation Center Södra Älvsborg, Sweden; c University of Gothenburg, Sahlgrenska Academy, Institute of Neuroscience and Physiology, Sahlgrenska Academy, University of Gothenburg, Sweden; d Region Västra Götaland, Research, Education, Development & Innovation Primary Health Care, Sweden; e Region Västra Götaland, Research, Education, Development & Innovation Primary Health Care, Research, Education, Development & Innovation Center Fyrbodal, Sweden; f Department of Social Work, University of Gothenburg, Gothenburg, Sweden; g Region Västra Götaland, Research, Education, Development & Innovation Primary Health Care, Research, Education, Development & Innovation Center Skaraborg, Sweden

**Keywords:** Sick leave, sense of coherence, health literacy, quality of life, health care economics and organizations

## Abstract

**Objective:**

To examine characteristics of primary healthcare patients with long-term impaired work ability, and to assess the correlation between sense of coherence and factors related to health, function, and work ability.

**Design and setting:**

Cross-sectional study including patients from the LEARN-to-COPE cluster-randomized controlled trial, conducted across 40 primary care centers in Region Västra Götaland, Sweden.

**Subjects:**

Primary healthcare patients with recurrent or long-term sick leave or health-related unemployment (*n* = 243).

**Data collection:**

Sick leave data were collected from the Swedish Social Insurance Agency. Demographics and contextual data were patient-reported or retrieved from personal identity numbers. Symptoms, health-related quality of life, health literacy, sense of coherence, perceived work ability, and lifestyle were assessed using validated questionnaires.

**Results:**

Mean age was 47.4 years. Most were women born in a Nordic country, had at least secondary education, and were gainfully employed. A third was unemployed. Mean number of sick days was 1,215 (SD 1,010), and 67.9% were on full-time sick leave. Perceived work ability was low. Participants reported severe anxiety and exhaustion, moderate depression, and a high risk of long-term sick leave due to pain. Health-related quality of life was extremely low. Half reported inadequate or problematic health literacy, and sense of coherence was low. Smoking and obesity were common, physical activity levels were average, and excessive alcohol consumption was below average. About half participated in any rehabilitation activities. Sense of coherence was significantly correlated with health literacy, health-related quality of life, symptoms of mental illness, perceived work ability, and pain (all *p* < 0.001); but not with sick leave duration or participation in rehabilitation.

**Conclusion:**

Given participants’ pronounced suffering, improving health-related quality of life among primary healthcare patients with long-term impaired work ability should be prioritized. Sense of coherence was associated with several determinants of sick leave, but not with its previous duration.

**Trial registration number:**

clinicaltrials.gov NCT04254367.

## Introduction

Long-term sick leave represents a growing challenge in Sweden. While the annual incidence of new sick-leave spells exceeding 30 days has remained stable at approximately 60 per 1,000 inhabitants, the average duration has increased from 148 to 166 days, resulting in an 18% increase in ongoing cases. Recent statistics show that about 50% of sick leave cases lasting 30 days or more were closed within three months, another 20% within six months, and an additional 15% within one year. Approximately 94% were closed within two years [1]. Very long-term sick leave cases are rare, but they have a significant impact on the average duration of sick leave. The majority of sick leave certification is effectuated in primary care.

While several qualitative studies have explored the experiences of primary healthcare patients on long-term sick leave, including those with common mental disorders and chronic pain, few studies have addressed the issue of horizontal prioritization between these patient groups and others. Moreover, our understanding of the actual health problems faced by primary care patients – beyond what is captured in diagnostic codes – remains limited. A more nuanced characterization of patients’ symptoms and coping abilities, complemented by correlation analyses, could help tailor interventions for this group and facilitate meaningful prioritizations.

This paper aims to deepen the understanding of health complaints among primary care patients on long-term sick leave and to inform appropriate responses, priorities, and interventions for this frequently attending yet often overlooked patient group. Additionally, the study contributes to understanding possible correlations between sense of coherence and factors related to health, functioning, and work ability.

The relationship between long-term sickness absence, disease, and illness is not yet fully understood. The number of long-term sick leave cases covaries with the number of sick leave cases started [2], but an increase in sickness absence does not necessarily correspond to a rise in health complaints. For instance, in Norway, the prevalence of somatic complaints remained stable at a time when sick leave surged by 65% [3], and interventions reducing symptoms and increasing well-being do not necessarily entail lower sickness absence rates [4].

A proposed explanation for the growing sickness absence despite stable health complaints is that demographic, condition-specific, individual, and organizational factors all contribute to the risk of long-term sick leave [5]. Soft factors, like workers’ intention to return to work [6,7], beliefs about recovery, a sense of control, fear-avoidance and perceiving the health issue as work-related may impact work participation [6]. Frontline employees from different authorities agree that it is important to reach consensus on a person-centered plan to address identified non-medical practical problems alongside medical care [8]. However, the Swedish sick leave and rehabilitation process (SRP), when applied in the primary care context, is perceived to create a situation in which the patient loses control [9].

Patients’ narratives of the SRP often reveal profound struggles with reduced work capacity due to health issues, making the prospect of returning to work feel nearly impossible [10–12]. Although many SRP patients attempt to return to work, they may discontinue due to their health conditions, fear of overexertion, personal circumstances, or inadequate employer support [10,11,13,14].

From a professional standpoint, the most challenging SRP cases involve persistent or recurrent, elusive or complex conditions for which no simple solutions exist to alleviate the patient’s suffering [4]. There is a risk that repeated consultations due to persistent ill-being may lead to referral loops, inadequate condition management, and increased patient frustration [15].

Frequent attendance as a phenomenon, whether transitory or persistent, has been linked to elevated work absence rates, more frequent sick leave episodes, extended sick leaves, and disability pensions [16]. Frequent attenders as a group have reduced health-related quality of life in all domains [17,18] and exhibit a higher prevalence of psychiatric and somatic diagnoses compared to non-frequent attenders [19]. Pain and psychosocial distress are the most common complaints of frequent attenders [17], and clinical complexity often increases due to multimorbidity involving both somatic and mental health conditions [18].

Evidence suggests that more than half (56%) of frequent healthcare attendance cannot be explained solely by biomedical factors [20]. Psychosocial and demographic predictors include low education, high health anxiety [18], panic disorder, impaired coping strategies [21,22], poor self-rated health, reduced physical functioning, younger age, unemployment [23], and social isolation [24]. Approximately 15–20% of frequent attenders remain persistently so over several years [25]. Multifactorial contributors to frequent attendance are still often underdiagnosed [26].

Sense of coherence, reflecting the perception of life as comprehensible, manageable, and meaningful, serves as a protective factor against the adverse effects of stressors, including illness [27]. A low sense of coherence is associated with characteristics observed in patients with persistent physical symptoms and long-term sick leave, including poor illness adjustment, increased risk of comorbid depression [28], and frequent healthcare utilization [22]. Moreover, low sense of coherence has been shown to predict prolonged sick leave [29–34]. Among individuals on sick leave, it tends to be strong among independent SRP patients motivated to return to work despite persistent symptoms, low among help-seeking, ambivalent patients, and moderate among those who have ceased striving to return to work and adapted to their circumstances [35].

We know that soft factors influence the need for sick leave and frequent attendance, and that sense of coherence serves as a protective factor against the adverse effects of stressors, including illness. Nevertheless, our understanding is not yet sufficient to translate this knowledge into clinical practice. Assessing the correlation between sense of coherence and factors related to health, functioning, and work ability is essential for evaluating the potential effects of interventions targeting sense of coherence.

The purpose of this cross-sectional study was to examine the characteristics of primary healthcare patients with recurrent or long-term sick leave, or with health-related unemployment, to improve understanding of the target group. The study also aimed to assess the correlation between sense of coherence and factors related to health, function, and work ability.

## Materials and methods

### Trial design

This cross-sectional study was conducted as part of the LEARN-to-COPE cluster-randomized controlled trial [36], compiling and analyzing demographics, baseline values, and contextual data for included patients. The LEARN-to-COPE study aimed to evaluate the LEARN-to-COPE intervention, designed to strengthen participants’ sense of coherence and health literacy, with the aim of improving their work ability, well-being, and coping. The LEARN-to-COPE study was preregistered in ClinicalTrials.gov, Study ID NCT04254367.

### Setting

Data collection was conducted between autumn 2019 and autumn 2021 across 35 primary care centers (PCCs) in Region Västra Götaland, Sweden. Region Västra Götaland is a large region with approximately 1.8 million inhabitants, encompassing both urban and rural areas, patient populations with diverse care needs, and PCCs of varying sizes staffed by general practitioners, nurses, and psychosocial teams. Half of included PCCs were public and half private, although all were publicly funded.

### Participants

All patients enrolled in the LEARN-to-COPE cluster randomized controlled trial were included in this cross-sectional study. The enrollment of participants has been described in a previous article [36]. LEARN-to-COPE participants were primary healthcare patients of working age (18–64 years) who were experiencing long-term or recurrent health-related sick leave or unemployment, resulting in more than 60 net sick days during the last six months, regardless of diagnosis. Patients who were expected to return to work or job-seeking following an existing healthcare and rehabilitation plan were excluded, as were patients with severe somatic or psychiatric disease, cognitive impairment or not speaking/understanding Swedish.

### Variables and data sources

Age and gender were obtained from personal identity numbers as no participants identified as a gender other than male or female, while marital status, country of birth, education level, and current occupation/job title were reported by the patients themselves. Duration of impaired work ability for unemployed participants was self-reported, whereas for those on sick leave, it was both self-reported and sourced from the MicroData for Analysis of Social Insurance (MiDAS) database. All participants self-assessed changes in their activity level during the current sick spell by answering the question, ‘Have you increased your activity level during the current sick spell? Yes/No’. The question referred to activity in a general sense, without specifying physical, social, or occupational activities.

Employment status was categorized using the Statistics Sweden socio-economic classification system, known as ‘Socioeconomic Indexation’ (SEI) [37]. Job titles were classified into three categories: high white collar, middle/low white collar, and blue collar/students [38].

Perceived work ability was evaluated using the single-item Work Ability Score (WAS), which asks respondents to rate their current work ability compared with their lifetime best on a scale from 0 to 10 [39,40]. Sense of coherence was measured with the Sense of Coherence Scale-13 (SOC-13) [27]. Health literacy was assessed using the European Health Literacy Survey Questionnaire, 16-item version, Swedish edition (HLS-EU-Q16-SE) [41]. Health-related quality of life was determined with the EuroQol 5-Dimension (EQ-5D) [42], which examines five areas: mobility, self-care, usual activities, pain/discomfort, and anxiety/depression, independent of any specific health condition.

Mental health symptoms, including depression, exhaustion, and anxiety, were measured using the Montgomery-Åsberg Depression Rating Scale -Self-rated version (MADRS-S) [43], the Karolinska Exhaustion Disorder Scale (KEDS) [44], and the Generalized Anxiety Disorder 7-item Scale (GAD-7) [45]. The risk of future disability and long-term sick leave due to pain was assessed with the Örebro Musculoskeletal Pain Screening Questionnaire (ÖMPSQ) [46]. The prevalence of widespread pain, defined as pain on both sides of the body, above and below the waist, and in the axial skeleton [47], was evaluated using predefined body regions [48]. Additionally, pain catastrophizing, a factor contributing to long-term debilitating pain, was measured with the Pain Catastrophizing Scale (PCS) [49].

The participants reported the medications they took daily, including prescribed drugs, over-the-counter medications, and natural remedies. The medications were manually analyzed for antidepressants, drugs recommended for long-term pain management, and prescription addictive medications.

Leisure-time physical activity was assessed using the Leisure Time Physical Activity Instrument (LTPAI) [50]. A sedentary lifestyle was defined as prolonged sitting or minimal exercise (not significantly increasing the breathing rate) for less than 4 h per week. Hazardous alcohol consumption, defined as 3–4 standard drinks corresponding to 12 grams of pure alcohol 2–3 times per week or more, was evaluated based on questions regarding frequency and volume of intake. Smoking included both daily and occasional use. Body Mass Index (BMI) was used to categorize patients as underweight, normal weight, or obese.

Validated questionnaires and questions about contextual data were distributed through secure web links and stored in esMaker (Entergate AB), connected to the researcher’s account on Region Västra Götaland’s protected network. The research assistant managed the distribution and sent reminders.

### Statistical methods

Participant identities were anonymized with unique codes prior to analysis. Questionnaire responses were compiled and interpreted according to the guidelines provided in each questionnaire’s manual. No subgroup analyses or interactions were explored.

Standard statistical methods were applied for descriptive analyses. Continuous variables were analyzed using independent-samples t-tests or Mann–Whitney U tests, and categorical variables and frequencies were analyzed using Pearson’s chi-square test.

Bivariate correlation analyses (Pearson’s r for parametric and Spearman’s ρ for nonparametric data) were used to assess correlations between sense of coherence and other continuous variables: health literacy, health-related quality of life, symptoms of mental illness, perceived work ability, participation in rehabilitation (self-reported, total hours), and net sick-leave days (all diagnoses, registry-based data). T-tests or one-way ANOVA were used to examine mean differences between sense of coherence and categorical variables, including risk of long-term sick leave due to pain, prevalence of widespread pain, pain catastrophizing, lifestyle habits, and participation in medical or work-oriented rehabilitation.

Linear regression analyses were conducted for the continuous outcome variables described above, while linear mixed-effects models were used to analyze categorical outcomes and to account for clustering due to randomization at the primary care center level. All analyses were adjusted for age, sex, allocation in the LEARN-to-COPE study, antidepressant use, educational level, employment status, and baseline physical activity.

All statistical analyses were performed using IBM SPSS Statistics, version 29. Statistical significance was set at *p* < 0.05. No adjustments were made for multiple testing.

The study results were reported in accordance with the STROBE checklist for observational studies [51].

## Results

Between January 2020 and September 2021, 243 participants from 35 PCCs, over half of the patients approached for consent, were enrolled in the LEARN-to-COPE study. This cross-sectional study analyzed the baseline data from all 243 participants. The participants were recruited from both urban and rural PCCs, as well as from small and large PCCs. Additionally, they originated from PCCs with varying Care Need Indexes, reflecting different socioeconomic contexts.

Personal identity numbers were available for all 243 participants. Registry data from the Social Insurance Agency were accessible for 215 participants (88%), although not complete for all, and baseline questionnaire and contextual data were available for 219 participants (90%). Some self-reported data are missing, particularly regarding work history and occupation, as these questions were occasionally left unanswered by participants.

### Descriptive data

Participant demographic characteristics and study variables at baseline, presented as valid percentages, are detailed in Tables 1 and 2. The participants were 24–64 years old (mean 47 years), and the majority were females. Most were born within a Nordic country. Only a few participants had primary education as their highest level of education, while the majority held either secondary education or a university/college degree. About two-thirds were employed, one-third were searching for work, and less than 1% were studying. Nearly one in ten was self-employed or hourly employed. The socioeconomic distribution based on profession indicated that participants were more likely to have either high white-collar jobs or low socioeconomic status jobs than middle-tier occupations. See Table 1.

**Table 1.** Demographic characteristics of primary healthcare patients aged 18–64 years experiencing long-term or recurrent health-related sick leave or unemployment, resulting in over 60 net sick leave days in the past six months, regardless of diagnosis – cross-sectional data from the LEARN-to-COPE cluster randomized controlled trial. Missing values not included. Age and gender were retrieved from registry data, whereas marital status, country of birth, education, work history, and occupation were self-reported. Total number of participants *N* = 243.

Demographic characteristic		Number of respondents N_total_ = 243
**Age years mean (SD)**		
**Gender, n (%)**	47.4 (9.7)	243
Women	175 (72.0)	243
Men	68 (28.0)	243
**Marital status, n (%)**		
Married/cohabiting/partnership	149 (68.0)	219
Single/divorced/widow/widower	70 (32.0)	219
**Born outside a Nordic country, n (%)**	19 (8.8)	216
**Educational level, n (%)**		
Up to primary education (nine years of compulsory schooling)	15 (6.8)	219
Secondary education	129 (58.9)	219
University or college	75 (34.2)	219
**Working history, n (%)**		
High white collar	55 (42.3)	130
Middle / low white collar	22 (16.9)	130
Blue collar / students	53 (40.8)	130
**Occupation, n (%)**		
Unemployed	62 (29.5)	210
Studying	1 (0.5)	210
Gainful employed	147 (69.7)	210
*Self-employed*	6 (2.9)	210
*Hourly employed*	16 (7.6)	210

**Table 2.** Work absence, symptoms, and coping among patients with long-term sick leave or health-related unemployment in primary healthcare. Missing values not included. Registry data retrieved from the MicroData for analysis of social insurance (MiDAS) database. Cross-sectional data from the LEARN-to-COPE cluster randomized controlled trial.

Study variable		Number of respondents N_total_ = 243
**Duration of impaired workability**		
*Net sick leave days, registry data, mean (SD)*	1215 (1010)	168
*Sick leave > 365 days, registry data, n (%)*	135 (81.8)	168
*Sick leave > 1000 days, registry data, n (%)*	113 (51.1)	168
*Part-time early retirees, registry data, n (%)*	13 (5.3)	168
*Health-related unemployment > 365 days, self-reported, n (%)*	49 (81.7)	60
**Self-reported level of sick leave and health-related unemployment**		
*Full-time sick leave, n (%)*	127 (67.9)	187
*Part-time sick leave 75%, n (%)*	8 (4.3)	187
*Part-time sick leave 50%, n (%)*	43 (23.0)	187
*Part-time sick leave 25%, n (%)*	9 (4.8)	187
**Self-assessed increase in activity level during current sick spell, n (%)**	105 (56.1)	219
**Perceived work ability (WAS), mean (SD)**	2.9 (2.7)	219
**Sick leave diagnoses, registry data**		
Psychiatric disorders/syndromes and behavioral disorders, n (%)	122 (62.2)	196
Musculoskeletal and connective tissue diseases, n (%)	36 (18.4)	196
Other diagnoses, n (%)	38 (19.4)	196
**Medication, n (%)**		
Antidepressant medication	109 (44.9)	219
Recommended drugs for long-term pain	56 (23.0)	219
Addictive drugs on prescription	36 (16.4)	219
**Symptoms of mental illness**		
Depression (MADRS-S), mean (SD)	21.0 (10.0)	219
Exhaustion (KEDS), mean (SD)	38.2 (9.6)	219
Anxiety (GAD7), mean (SD)	16.0 (5.6)	219
**Pain**		
Risk of long-term sick leave due to pain (ÖMPSQ), n (%)	113 (59.5)	190
Widespread pain, n (%)	89 (40.6)	219
Pain catastrophizing (PCS) > 30, n (%)	51 (23.3)	219
**Sense of coherence (SOC-13), mean (SD)**	51.4 (14.1)	219
Comprehensibility, mean (SD)	18.6 (6.2)	219
Manageability, mean (SD)	15.5 (4.8)	219
Meaningfulness, mean (SD)	17.3 (5.0)	219
**Health literacy (HLS-EU-Q16), mean (SD)**	12.0 (3.5)	219
Inadequate, n (%)	36 (16.4)	219
Problematic, n (%)	74 (33.8)	219
Sufficient, n (%)	109 (49.8)	219
**Health-related quality of life (EQ-5D), mean (SD)**	0.43 (0.3)	219
**Lifestyle habits**		
Sedentary lifestyle, n (%)	44 (20.1)	219
Alcohol high, n (%)	4 (1.8)	219
Smoking (yes + sometimes), n (%)	44 (20.1)	219
BMI > 30, n (%)	74 (34.9)	212
**Participation in any rehabilitation (self-reported), n (%)**	90 (48.1)	219
Medical rehabilitation, n (%)	70 (32.0)	219
*Weekly hours, mean (SD)*	3.0 (4.5)	219
Work-oriented rehabilitation, n (%)	33 (15.1)	219
*Weekly hours (independently of sick leave), mean (SD)*	5.1 (5.8)	219

**Figure 1.**
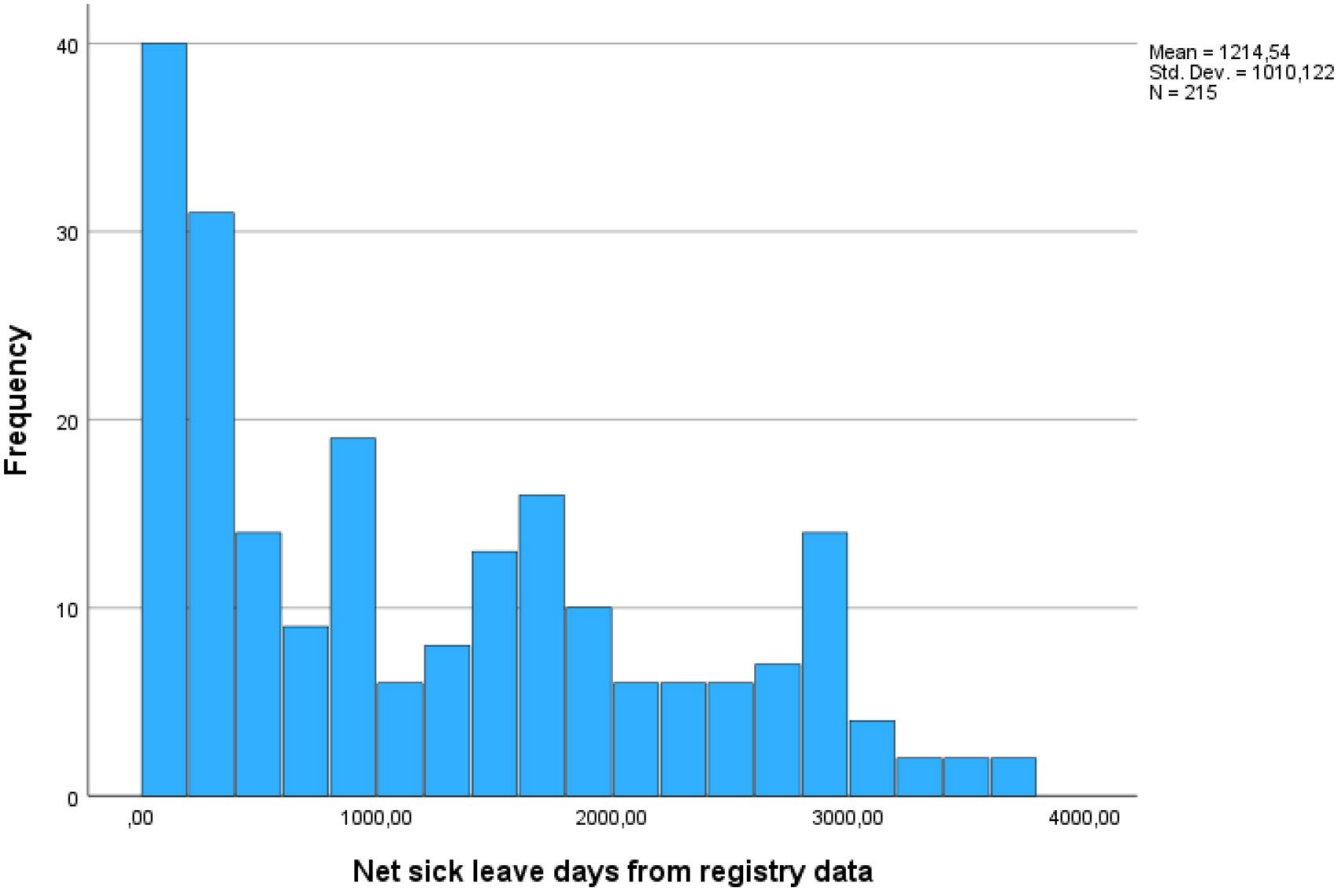
The histogram illustrates the distribution of the total number of net sick-leave days per individual in the study group. The mean number of net sick-leave days was 1,215, and the median was 943 days. The distribution was skewed distributed.

Most participants suffered from very long-term health-related impaired workability, and the majority were on full-time sick leave. On average, registered sick spells encompassed 244 net sick leave days in the year preceding baseline, calculated as full-time equivalents and thus including part-time sick leave, and 1,215 net days since the initiation of sick leave. The distribution of the total number of net sick leave days in the study group is shown in Figure 1. Additionally, most unemployed participants had experienced difficulties reestablishing themselves in the job market due to poor health for over a year.

Participants’ perceived work ability was very low, as shown in Table 2. When asked if they had increased their activity level since they were called off sick, just over half of the participants affirmed this. Moreover, only about half of the participants reported engaging in rehabilitation, with medical rehabilitation, such as physiotherapy or psychotherapy, being more prevalent than work-oriented rehabilitation. The number of scheduled rehabilitation hours was limited.

Assessment of sense of coherence revealed generally low scores across all subscales, indicating that participants perceived low levels of comprehensibility, manageability, and meaningfulness in their lives (see Table 2). Further, half of the participants exhibited inadequate or problematic health literacy.

Mental disorders were the most common causes of sick leave, followed by musculoskeletal disorders, and other diagnoses. Nearly half of the participants were prescribed antidepressants, one quarter were prescribed medications for long-term pain, and approximately one in six were prescribed addictive drugs.

Participants reported extremely low health-related quality of life. On the group level, participants self-assessed moderate symptoms of depression, pronounced symptoms of exhaustion, and severe anxiety. Further, more than half of the participants were at risk of long-term sick leave due to pain, a significant share met the criteria for widespread pain, and pain catastrophizing was common.

Self-reported unhealthy lifestyle habits were relatively common. One-third of participants had a BMI exceeding 30, thus defined as obesity. Additionally, one in five participants were smokers, and an equal proportion had a sedentary lifestyle. Very few participants reported hazardous alcohol consumption.

Health-related unemployment self-reported. Self-assessed change in activity level assessed with the question ‘Have you increased your activity level during current sick spell? Yes/No’. WAS: Work Ability Score; Antidepressant medication: non-addictive antidepressants; Recommended drugs for long-term pain: paracetamol, NSAIDs, chlorzoxazone, amitriptyline/nortriptyline, duloxetine, gabapentin; Addictive drugs: opioids, benzodiazepines, benzodiazepine analogues, codeine, pregabalin; MADRS-S: Montgomery-Åsberg Depression Rating Scale – Self-rated version; KEDS: Karolinska Exhaustion Disorder Scale; GAD-7: Generalized Anxiety Disorder 7-item Scale; ÖMPSQ: Örebro Musculoskeletal Pain Screening Questionnaire; Widespread pain was defined as pain on both sides of the body, above and below the waist, and in the axial skeleton. SOC-13: Sense of Coherence Scale-13; HLS-EU-Q16: European Health Literacy Survey Questionnaire, 16-item version; EQ-5D: EuroQol 5-Dimension; A sedentary lifestyle was defined as <4 h of light physical activity per week; hazardous alcohol consumption as ≥3–4 standard drinks 2–3 times per week or more; and smoking included both regular and occasional use. BMI = Body Mass Index.

### Correlation analyses focusing on sense of coherence and health-related quality of life

The correlation analyses revealed statistically significant positive correlations between sense of coherence and health literacy, health-related quality of life, and self-perceived work ability, as well as statistically significant negative correlations with symptoms of mental illness, pain catastrophizing, and the risk of long-term sick leave due to pain. However, no significant correlations were observed with net sick leave prior to this cross-sectional measurement, the prevalence of widespread pain, lifestyle habits, or participation in medical or work-oriented rehabilitation. See Tables 3 and 4 and Supplementary Information 1. Results were similar across the sense of coherence subscales. The correlations persisted after adjustment for confounders.

**Table 3.** Correlation analyses between sense of coherence (SOC-13) and continuous study variables among primary healthcare patients with long-term sick leave or health-related unemployment – cross-sectional data from the LEARN-to-COPE cluster-randomized controlled trial. Missing values not included. Total number of participants *N* = 243.

		Parametric correlations		Non-parametric correlations
Continuous study variable	Pearson correlation	*P*-value	Spearman’s rho	*P*-value
**Health literacy (HLS-EU-Q16)**	0.349**	**<0.001**	0.298**	**<0.001**
**Health-related quality of life (EQ-5D)**	0.355**	**<0.001**	0.356**	**<0.001**
**Symptoms of mental illness**				
MADRS-S (*n* = 219)	−0.702**	**<0.001**	−0.695**	**<0.001**
KEDS (*n* = 219)	−0.521**	**<0.001**	−0.510**	**<0.001**
GAD7 (*n* = 219)	−0.679**	**<0.001**	−0.673**	**<0.001**
**Perceived work ability (WAS)**	0.274**	**<0.001**	0.264**	**<0.001**
**Participation in any rehabilitation (self-reported), n (%)**				
Weekly hours of medical rehab	−0.103	0.397	−0.059	0.626
Weekly hours of work-oriented rehab	0.102	0.668	0.214	0.364
**Net sick leave days, all diagnoses, registry data**	−0.095	0.220	−0.091	0.239

^**^ Correlation significant at the 0.01 level (2-tailed). Bold text indicates statistically significant correlations with sense of coherence (p < 0.05). The significant results remained after adjustment for potential confounders. SOC-13: Sense of Coherence Scale-13; HLS-EU-Q16: European Health Literacy Survey Questionnaire, 16-item version; EQ-5D: EuroQol 5-Dimension; MADRS-S: Montgomery-Åsberg Depression Rating Scale – Self-rated version; KEDS: Karolinska Exhaustion Disorder Scale; GAD-7: Generalized Anxiety Disorder 7-item Scale; WAS: Work Ability Score; Participation in medical or work-oriented rehabilitation was self-reported. Net sick leave data was retrieved from the MicroData for Analysis of Social insurance (MiDAS) database.

**Table 4.** Results from t-tests, ANOVA, and Mann–Whitney analyses of mean differences between sense of coherence (SOC-13) and selected categorical variables among primary healthcare patients with long-term sick leave or health-related unemployment – cross-sectional data from the LEARN-to-COPE cluster randomized controlled trial.

	T-test/ANOVA				Mann-Whitney U	
Categorical study variable	Mean SOC-13	Std. Deviation	Std. Error Mean	*P*-value	Cohen’s d 95% CI	*P*-value
**Health literacy (HLS-EU-Q16)**						
Inadequate	42.6	13.0	2.2	**<0.001**		**<0.001**
Problematic	50.0	13.6	1.6			
Sufficient	55.2	13.3	1.3			
**Pain**						
ÖMPSQ < 50	57.0	12.9	1.5	**<0.001**	0.706	**<0.001**
ÖMPSQ ≥ 50	47.4	14.1	1.3			
Widespread pain, No	52.0	14.1	1.2	0.475		0.421
Widespread pain, Yes	50.6	13.9	1.5			
PCS ≤ 30	54.6	13.0	1.0	**<0.001**	1.084	**<0.001**
PCS > 30	40.8	12.1	1.7			
**Lifestyle habits**						
Sedentary lifestyle, No	51.8	14.2	1.1	0.357		0.253
Sedentary lifestyle, Yes	49.6	13.6	2.1			
Alcohol high, No	51.2	13.9	.95	0.191		0.246
Alcohol high, Yes	60.5	19.6	9.8			
Smoking, No	52.1	14.0	1.1	0.128		0.180
Smoking, yes + sometimes	48.5	14.0	2.1			
BMI < 30	51.5	14.1	1.2	0.973		0.877
BMI ≥ 30	51.5	14.2	1.7			
**Participation in any rehabilitation (self-reported), n (%)**						
Medical rehabilitation, No	52.1	13.7	1.1	0.285		0.331
Medical rehabilitation, Yes	49.9	14.9	1.8			
Work-oriented rehab, No	51.9	14.1	1.0	0.172		0.136
Work-oriented rehab, Yes	48.3	13.2	2.3			

^**^ Bold text indicates p < 0.05. The significant results remained after adjustment for potential confounders. Missing values not included. Total number of participants *N* = 243. A score of ≥50 on the ÖMPSQ indicates risk of long-term sick leave due to pain, widespread pain was defined as pain on both sides of the body, above and below the waist, and in the axial skeleton, and a score of >30 on the PCS indicates high pain catastrophizing. A sedentary lifestyle was defined as <4 h of light physical activity per week; hazardous alcohol consumption as ≥3–4 standard drinks 2–3 times per week or more; and smoking included both regular and occasional use. BMI = Body Mass Index. BMI values ≥30 are classified as obesity.

Furthermore, a statistically significant negative correlation was found between health-related quality of life and the number of net sick leave days during the year preceding baseline, independent of diagnosis (Pearson’s r = −0.253, *p* < 0.001; Spearman’s ρ = −0.273, *p* < 0.001). This correlation also persisted after adjustment for confounders. (The data are not presented in any table.)

## Discussion

Findings from this cross-sectional study showed that this cohort of primary healthcare patients of working age (18–64 years) who were experiencing long-term or recurrent health-related sick leave or unemployment, self-reported extremely low health-related quality of life and perceived workability, despite their non-urgent health status. Though their impaired workability lasted over 365 days for more than 80% of both unemployed and sick-listed individuals, only about half reported a self-assessed increase in their overall activity level during the current sick leave, and participation in rehabilitation.

Demographic data revealed that the participants were predominantly middle-aged females who were in a relation, and few were born outside a Nordic country. Most had at least secondary education, they were more likely to hold high white-collar or low socioeconomic status jobs than middle-tier occupations, and about a third were unemployed.

Participants also scored very low on sense of coherence. Analyses showed strong correlations between sense of coherence and factors related to perceived health, functioning, and work ability, but no correlations between sense of coherence and previous net sick leave, prevalence of widespread pain, lifestyle habits, or participation in rehabilitation. However, a significant negative correlation was found between health-related quality of life and previous net sick leave.

### Strengths and limitations

A key strength of this cross-sectional study is the comprehensive data collected from patients on long-term sick leave across diverse PCCs and contexts. This extensive dataset enabled the study to provide new insights into primary healthcare patients experiencing long-term or recurrent health-related sick leave or unemployment. The use of registry data and validated questionnaires focusing on current conditions enhanced data quality and reduced the risk of recall and information bias, thereby strengthening the study’s internal validity and reliability.

A limitation of the study is its cross-sectional design and lack of a control group, which warrant cautious interpretation of the findings. However, the results reveal patterns and explore correlations within the dataset, which may inform and guide future research.

Implementing clearly defined inclusion and exclusion criteria, along with recruiting participants from diverse PCCs, helped minimize the risk of selection bias. The inclusion criteria allowed for broad participation, which is appropriate for a pragmatic study conducted in primary care. However, the inclusion of both sick-listed and unemployed individuals resulted in a heterogeneous sample. At the same time, the study population mainly comprised patients with prolonged work absence, leading to limited representation of those with moderate-duration sick leave.

Notably, nearly half of the approached patients declined to participate in the LEARN-to-COPE study (*n* = 227, 48%) [36], which suggests a potential selection bias that should be considered when interpreting the results. Furthermore, the dataset is incomplete, as registry data from the Social Insurance Agency are unavailable for unemployed participants, and some self-reported data are missing. We do not know the specific reasons why some patients chose not to participate, but we know that patients who did not complete all questionnaires during the LEARN-to-COPE study often reported that the questionnaires were too comprehensive, that they had competing ‘must-do’ tasks, or that poor health or fatigue made it difficult for them to fully answer all questions. These reports indicate that non-responders may have been in similarly poor health as the responders, but the results may have differed with a different sample or response rate.

Additionally, five PCCs withdrew from the LEARN-to-COPE cluster-controlled study before participant inclusion [36]. One withdrew due to having an elderly patient population, and four due to a lack of time during the COVID-19 pandemic. We have no reason to suspect that patients from the withdrawn PCCs differed from those included, but this cannot be confirmed.

While the findings may be applicable to other primary healthcare patients on very long-term sick leave, the generalizability of the results depends on the specific context.

### Interpretation of results and comparison with other studies

Participants’ perceived work ability (WAS) was significantly below the mean value for the general Swedish population (WAS = 8.25) and for Swedish individuals on long-term sick leave, defined as having more than 90 gross sick leave days per year (WAS = 6.67) [52]. This was expected, as the most severe cases are expected to be filtered out over time. However, previous findings suggest that patients’ loss of hope resulting from a dysfunctional SRP may partly explain their low perceived work ability [9], highlighting the need for a more thorough analysis.

The participants rated their symptoms of depression, exhaustion, generalized anxiety, and pain on a level indicating most of the participants experiencing a complex mix of debilitating symptoms, in line with a low EQ-5D index. Nevertheless, it was unexpected that our primary healthcare cohort rated quality of life lower than patients with severe somatic diseases such as heart failure (EQ-5D index 0.49), schizophrenic disorder (EQ-5D index 0.59), or malignant neoplasm colon (EQ-5D index 0.68) [53]. Participant EQ-5D scores were also lower than other vulnerable cohorts: primary healthcare patients with depression (EQ-5D index 0.58) [54], and homeless people (EQ-5D index 0.73) [55].

We interpreted that though the participants did not suffer from a life-threatening disease, comorbidity with combined physical and psychiatric symptoms, multiple functional limitations, and low sense of coherence had severe impact on participants’ health-related quality of life, and their ability to gather the strength to change their situation. Like previous qualitative SRP findings [8,56], this study highlighted the complexity of the target group’s health problems, implying the need for coordinated support to enable improving SRP patients’ health, and function.

According to the Swedish Health and Medical Services Act, patients with the greatest needs should be given priority for available healthcare resources. This is typically interpreted as prioritizing patients with acute or severe medical conditions. The present study challenges this interpretation, suggesting that common primary healthcare patients may warrant as high a priority for healthcare resources as secondary care patients, based on the severity of their suffering.

Frontline employees in the SRP have reported that numerous factors beyond patients’ medical conditions influence the process’s outcomes [8]. In this study, we found that participants as a group were not uniformly more vulnerable than the general population in terms of health literacy, lifestyle habits, educational level or working history. Half of the participants showed inadequate or problematic health literacy at baseline, which indicated that half might make suboptimal health-related decisions due to an impaired ability to obtain, understand and act on health-related information, but this level of health literacy is average compared to the European reference population [57], as impaired health literacy is common. Further, comparing participants’ lifestyle habits to data from the Swedish Public Health Authority [58] revealed no difference in sedentary lifestyle, and the number of participants reporting excessive alcohol consumption was far below average, though smoking and obesity were twice as common among participants compared to the reference population. Finally, a significant share of participants held university or college degrees and had a work history in high-level white-collar positions. We conclude that complex health problems can affect anyone given unfortunate circumstances.

Despite the participants’ great suffering, this study highlighted that few participants were subjects of extensive interventions or support. Although the cohort had been on sick leave for over 1,200 days on average, likely involving repeated consultations and frequent attendance, only half reported participating in any rehabilitation. This implies that the other half received limited healthcare, primarily medication, and passive waiting.

Further, this study suggests that primary care patients with health conditions affecting their work ability are often not prioritized until late in the process. The selection criteria for the LEARN-to-COPE RCT included having more than 60 net sick days in the past six months regardless of diagnosis and occupational status, lacking a clear healthcare and rehabilitation plan to facilitate return to work, and not having severe diseases or cognitive impairments. Nevertheless, although the intention was to enroll patients as soon as a prolonged sick spell was suspected, the enrolled participants had an average sick spell duration of 1,200 days. Hence, our study indicates that primary healthcare employees tended to prioritize enrolling very long-term and complex SRP cases over shorter-term cases with better prognoses. This finding aligns with reports from frontline employees across various professions – physiotherapists, occupational therapists, psychotherapists, psychologists, general practitioners, and social insurance administrators – who all perceived that they were involved in patient cases too late, thereby limiting the effective use of their professional expertise and negatively impacting patient outcomes [56].

Sense of coherence among participants was −1.6 SD compared to a Swedish reference population [59], and the correlation analyses showed statistically significant positive and negative correlations in line with sense of coherence predicting health and sick leave, as expected based on prior research [22,28–34].

The study design did not allow determination of the direction of correlations or causal inference. However, we suggest that reciprocal correlations between sense of coherence and the other variables are plausible. Patients’ sense of coherence – their ability to understand and manage symptoms while maintaining a sense of meaning despite persistent symptoms – may influence health-related quality of life, symptoms, self-perceived work ability, and health literacy, which in turn may affect their sense of coherence.

If sense of coherence is not entirely determined by the other variables, the correlations identified would highlight the importance of supporting patients’ sense of coherence alongside standard medical treatment. Such support could include patient education aimed at enhancing symptom understanding, self-care, and context-specific problem-solving, in line with previous findings [8].

Interestingly, sense of coherence showed no significant correlation with participation in medical or work-oriented rehabilitation. This lack of correlation is noteworthy, as the aim of rehabilitation in the SRP context – medical rehabilitation, patient education, and work-oriented rehabilitation – is to help patients understand their symptoms (enhancing comprehensibility), improve symptom management (enhancing manageability), and engage in meaningful activities (enhancing meaningfulness) to strengthen their health and work ability.

However, the lack of correlation aligns with previous research highlighting both a perceived shortage of suitable interventions for patients on sick leave and the absence of regulations enabling coherent SRP governance [56]. We argue that if participation in rehabilitation is not clearly grounded in a realistic, person-centered plan addressing the biopsychosocial barriers to patients’ health and work ability, there is a risk that patients will perceive the interventions as incomprehensible, unmanageable, and meaningless, resulting in a loss of control. Further research is needed to explore how sense of coherence can be strengthened in practice.

There was no statistically significant correlation between sense of coherence and net sick leave. This finding aligns with previous research indicating that sense of coherence fluctuates rather than declines during the course of sick leave [35]. However, we found a statistically significant negative correlation between health-related quality of life and net sick leave the year preceding the study. Interestingly, this correlation remained significant after adjusting for potential confounders, including antidepressant use (as a proxy for symptom burden), educational level and employment status (as proxies for socioeconomic burden), and physical activity (as a proxy for lifestyle). Therefore, since patients’ coping ability – reflected by their sense of coherence – did not correlate with sick leave duration, another, unidentified factor appears to contribute to prolonged sick leave. Based on previous research highlighting the lack of practical support and continuity of care for SRP patients, we suggest that a loss of control following a suboptimal SRP may represent this contributing factor [9,36,56]. This interpretation aligns with prior research, which has found that sense of coherence correlates with control-related factors – such as job control, psychosocial conditions, and social support – rather than with specific sociodemographic characteristics [60].

In conclusion, this study highlights that there is room for improvement in the care provided to primary care patients with impaired work ability and that this patient group warrants prioritization based on the severity of their suffering. Further, it supports earlier studies emphasizing the importance of a timely (early) plan for investigation, care, and rehabilitation in the SRP to prevent the adverse effects of being on sick leave while optimizing health outcomes [8,9,61]. Finally, it suggests that fostering a sense of coherence may improve target group symptoms and health-related quality of life. Potentially, focusing on care delivered with overall responsibility for the patient from the start, along with rehabilitative interventions aimed at enhancing patients’ sense of coherence – rather than repeatedly moving patients through standardized rehabilitation programs without considering their individual needs and values – could lead to improved sense of coherence and SRP outcomes. It may not be the patients’ sense of coherence itself that matters, but rather the SRP system’s capacity to enhance it.

There is a saying, often attributed to W. Edwards Deming: ‘Every system is perfectly designed to get the results it gets’. In this light, we interpret the organization of Swedish primary healthcare as one that leads employees to prioritize very long-term and complex SRP cases over shorter-term cases with better prognoses, potentially resulting in adverse outcomes. In line with previous research [56], we argue that the timing of interventions within the SRP warrants closer scrutiny, supported by organizational priorities that foster coherent process governance, interprofessional collaboration, overall liability, and continuity of relations.

### Clinical implications

This cross-sectional study, combining descriptive data, correlation analyses, and contextual interpretation, underscores the need for primary healthcare to be organized and resourced to prioritize patients whose health conditions impair their work ability. In addition, it suggests the importance of ensuring not only adequate medical treatment but also individualized care that supports patients’ sense of coherence. Future research should investigate how early consensus on each patient’s needs can be achieved and how SRP coordination across professional and organizational boundaries can be strengthened to promote consistent communication and enhance patients’ sense of coherence.

## Conclusion

This cross-sectional study found that Swedish primary healthcare patients experiencing recurrent or long-term health-related sick leave or unemployment reported health-related quality of life comparable to patients with severe cancer, attributed to a complex combination of mental and musculoskeletal symptoms. We also found statistically significant correlations between sense of coherence and factors related to health, function, and work ability that warrants further studies. These findings underscore the need for greater attention to this patient group, despite their non-urgent health status, including consideration of their sense of coherence.

## Data Availability

Complete data cannot be made publicly available for ethical and legal reasons according to the Swedish regulations of the ‘Act concerning the Ethical Review of Research Involving Humans (2006:460)’ (https://www.kliniskastudier.se/english/for-researchers/laws-regulations/act-concerning-ethical-review-research-involving-humans–.html) and the Swedish Ethical Reviews Authority (https://etikprovningsmyndigheten.se). Data are, however, available from the corresponding author on reasonable request.
